# Psoas hitch procedure in 166 adult patients: The largest cohort study before the laparoscopic era

**DOI:** 10.1002/bco2.85

**Published:** 2021-04-06

**Authors:** V. H. Groen, M. T. W. T. Lock, I. B. de Angst, P. C. M. S. Verhagen, S. Horenblas, P. Dik, J. L. H. R. Bosch

**Affiliations:** ^1^ Department of Urology University Medical Centre Utrecht Utrecht The Netherlands; ^2^ Department of Urology Erasmus University Medical Centre Rotterdam Rotterdam The Netherlands; ^3^ Department of Urology The Netherlands Cancer Institute Amsterdam The Netherlands; ^4^ Department of Pediatric Urology University Medical Centre Utrecht Utrecht The Netherlands; ^5^ Department of Pediatric Urology Sechenov University Moscow Russia

**Keywords:** psoas hitch procedure, treatment outcome, ureter, ureteral injury, ureteral obstruction, ureteral reimplantation

## Abstract

**Objectives:**

To present the short‐term and long‐term outcomes of the psoas hitch procedure in a large cohort with long‐term follow‐up.

**Patients and methods:**

A multicenter, retrospective cohort study was conducted. Patients were included if they had undergone an open psoas hitch procedure with ureteral reimplantation for different types of distal ureteral pathology between 1993 and 2017. Clinical failure was defined as radiologically‐proven obstruction of the ureteroneocystostomy and/or post‐operative complaints requiring additional surgery. Pre‐operative demographic data and post‐operative radiological imaging were collected. Complications were categorized as peri‐operative, acute (<30 days), and long‐term complications.

**Results:**

A total of 166 patients had undergone a psoas hitch procedure, with a median follow‐up of 15 months (IQR 6‐45). Indications for the procedure included intra‐operative injury of the ureter during gynecological, urological or general surgery, transitional cell carcinoma of the distal ureter, fistulae, (radiation) fibrosis, and trauma. There was no significant difference in pre‐ and post‐operative estimated glomerular filtration rate. Post‐operative complications included urinary leakage, recurrent urinary tract symptoms, recurrent malignancy, and kidney failure. Postoperative imaging was available in 143 patients. Failure of the psoas hitch procedure was seen in 8% (11/143) of the patients. In 55% (6/11) of these patients, radiation fibrosis was the indication for the psoas hitch procedure.

**Conclusion:**

This study provides greater insight into the long‐term complications of the open psoas hitch procedure in adults. The psoas hitch procedure can be considered a safe procedure for restoring the continuity of the ureter for different types of ureteral pathologies in adult patients. However, patients with a history of radiation therapy causing retroperitoneal fibrosis might be more prone to failure after the procedure.

## 1 INTRODUCTION

The psoas hitch procedure is a surgical technique that was first described by Witzel in 1896.^1^ Zimmerman et al. reported the first case series in 1960.^2^ In 1968, the procedure was adjusted by Harrow, who added a subepithelial tunnel technique to prevent reflux,^3^ and it was then named the “psoas hitch procedure” by Turner‐Warwick and Worth.^4^ Over the last few decades, the procedure has become a popular technique to bridge the distal third of the ureter. The psoas hitch procedure has some benefits compared to other ureteral‐bridging techniques. Alternative techniques to restore the continuity of the ureter are the Boari flap procedure, intestinal interposition, transureteroureterostomy, cutaneous ureterostomy, and autotransplantation of the kidney.^5^ An advantage of the psoas hitch procedure is the use of native bladder instead of intestinal interposition, thus preserving urothelial continuity, an uncompromised blood supply, and preventing post‐operative complications such as urinary tract infections, metabolic abnormalities, mucus, and stone formation.

Indications for the psoas hitch procedure are (iatrogenic) ureteral injury,^3^ resection of a distal ureteral tumor, ureteric obstruction, and ureteral fistulae secondary to pelvic surgery or radiotherapy of the lower abdomen. Contraindications for the psoas hitch procedure are scarce. Severe hypertrophy of the bladder wall and previous extensive lower abdominal surgeries are tricky to proceed to this operation. Other relative contraindications are radiation of the lower abdomen, urethral strictures, neurogenic bladder, and bladder neck obstruction.^6^

The psoas hitch procedure has been described in a few combined (children and adults) case series, the majority dating from 1969 to 1984.^5^, ^17^ The psoas hitch procedure has shown to be an effective technique to restore ureterovesical continuity with success rates ranging from 72% to 96.7%, follow‐up ranging from 17 months up to a mean follow‐up of 4.5 years, and with minimal complications.^11^, ^14^, ^15^ In 2011, we reported a smaller case series of 33 patients who had undergone a psoas hitch procedure in two large university medical centers in the Netherlands. Surgical success was seen in 93.9% (31/33) of the patients with a follow‐up of 3‐189 months.^18^ The aim of the present study is to report long‐term results, including the clinical failure of the psoas hitch procedure, in a large retrospective cohort of adult patients.

## 2 PATIENTS AND METHODS

### 2.1 Patients and methods

A multicenter, retrospective study was performed after obtaining approval from the Institutional Review Boards of the participating hospitals (reference number WAG/mb/17/024269). Informed consent was waived because of the retrospective review of records. The records of 166 patients who underwent a psoas hitch procedure at the University Medical Center Utrecht and Erasmus University Medical Center from 1993 to 2017 were reviewed.

### 2.2 Surgical technique

**FIGURE 1:**
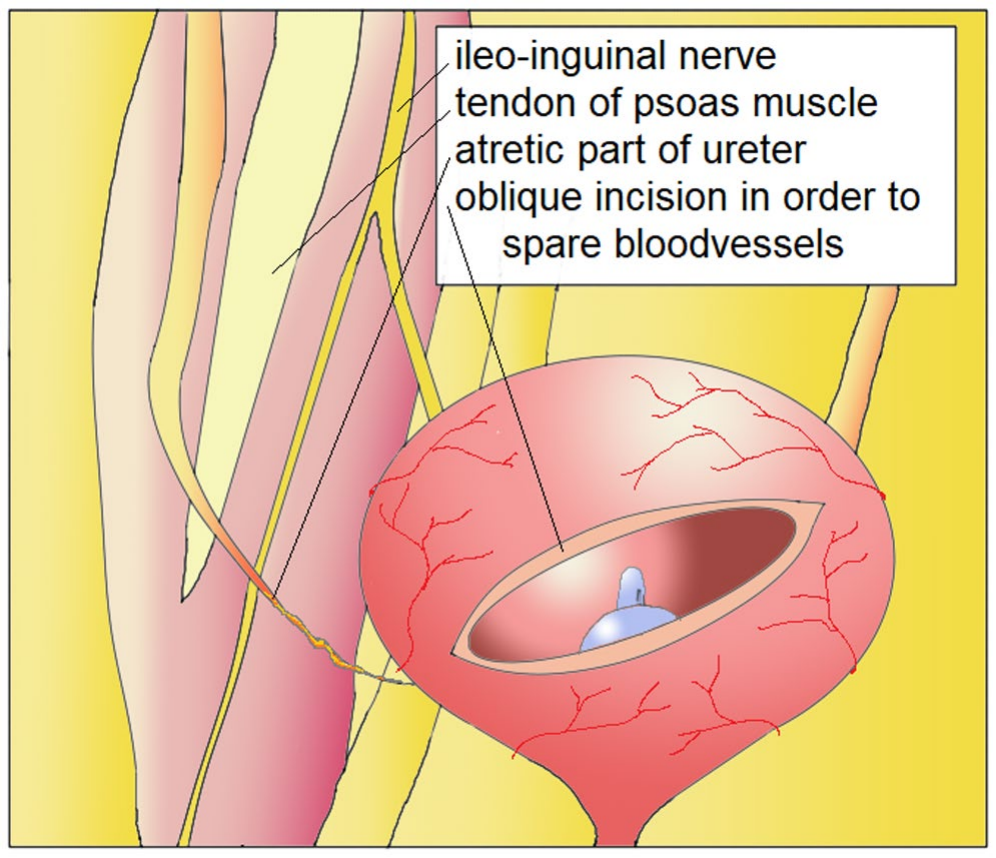
Mobilization of bladder dome, transection of urachus. Psoas muscles exposed. Ilioinguinal nerve identified. Oblique incision in order to spare blood vessels

**FIGURE 2:**
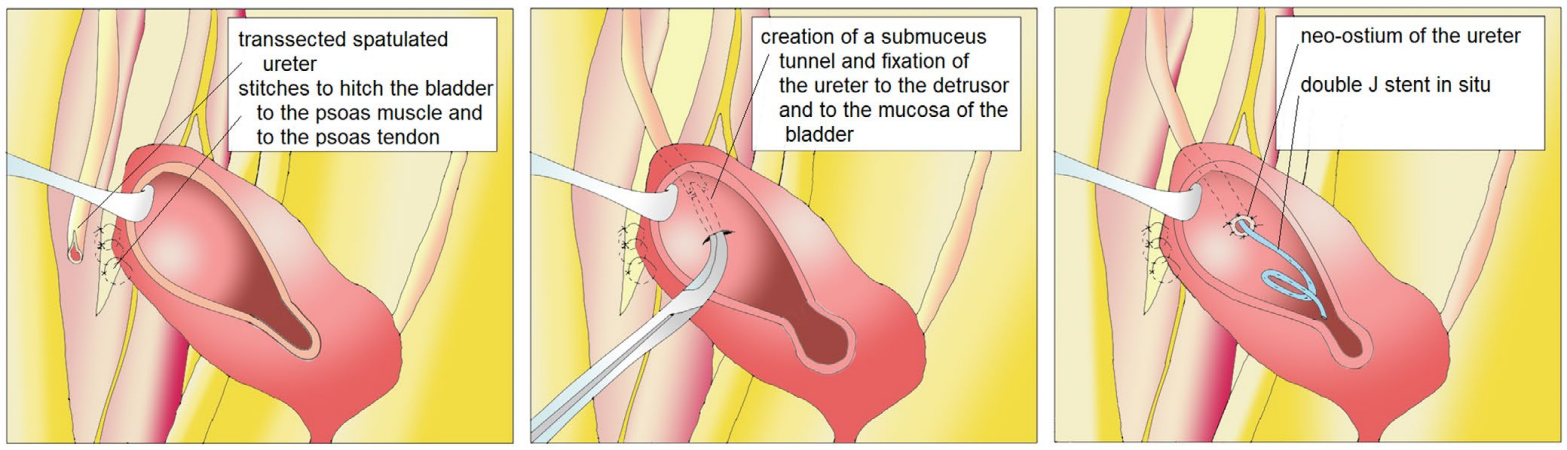
Atretic and obstructing part of ureter removed, fixation of the bladder to psoas muscles and to the tendon of the psoas muscle, creation of a submucosal tunnel, fixation of the ureter into the bladder wall, leaving a double J stent in situ

The surgical technique has previously been described by Turner‐Warwick and Worth.^4^ First, a Pfannenstiel or lower abdomen incision is made, then the ureter is identified and the extent of the ureteral pathology is assessed. Next, the psoas minor tendon must be identified above the level of the iliac vessels. The bladder is mobilized from the peritoneum with the division of the contralateral obliterated umbilical artery and, if necessary, bilateral division. The bladder is then opened, after being filled with 200‐400 cc, transversely and laterally to the bladder dome and moved upwards to the affected ureter and hitched to the psoas minor tendon. Caution is warranted not to include the genitofemoral nerve. Ureteral reimplantation is preferably performed using a tunnel technique. A splint or double‐J catheter is placed in the reimplanted ureter. Finally, the bladder is closed in an oblique‐longitudinal fashion (Figures 1, 2).

### 2.3 Outcome measures

Clinical failure, defined as radiologically‐proven obstruction of the ureteroneocystostomy and/or post‐operative complaints requiring additional surgery, was the primary outcome. Post‐operative radiological imaging includes antegrade pyelography (X‐APG), ultrasonography, computed tomography (CT), computed tomography intravenous pyelography (CT‐IVU), renography, and micturition cystography. Some patients had undergone previous procedures; these procedures were classified as conservative treatment i.e. percutaneous nephrostomy (PCN) and double‐J stenting, endo‐urological procedures requiring ureteroscopy (URS), or complex surgical procedures such as previous ureteral reimplantation and Boari‐flap procedures. Pre‐ and post‐operative renal function is given as serum creatinine level and estimated creatinine clearance according to the method of Cockcroft and Gault.^19^

Complications were categorized as peri‐operative, acute (<30 days) and long‐term complications (>30 days). Acute complications were retrospectively graded according to the Clavien‐Dindo classification.^20^ Long‐term complications were classified as urinary incontinence, hydronephrosis, ureteral stricture, infections, and renal failure. If applicable, procedure‐related re‐interventions and mortality were reported. To assess the primary outcome, we included patients who were considered to provide sufficient data to radiologically assess clinical failure. In these patients, at least one type of post‐operative imaging was available. The available post‐operative imaging had mostly been indicated for follow‐up of the underlying disease (i.e., CT‐scan to assess gynecological tumor response). These post‐operative imaging reports were considered valid to assess the radiological passage of the ureteroneocystostomy, as long as the condition of the kidneys and ureters were mentioned in the imaging reports. We used a pragmatic approach and created a hierarchical order of certainty with which a post‐operative imaging type can prove the radiological passage of a ureteroneocystostomy. The types of imaging were arranged in the following order: X‐APG, renography, CT‐IVU, CT‐abdomen, and ultrasound.

### 2.4 Statistical methods

Descriptives were used to display baseline and clinical characteristics, as well as complication rates. Continuous variables were displayed by the mean and standard deviation if normally distributed or by median and range if not normally distributed. A paired‐samples t test was used to determine a statistical significance of differences between pre‐ and post‐operative renal function, with a *P*‐value < .05 considered statistically significant. All analyses were conducted using the IBM Statistical Package for the Social Sciences, version 25.0 (SPSS Inc., Chicago, IL, USA).

## 3 RESULTS

### 3.1 Patients and treatment characteristics

A total of 166 patients, 43 (26%) men and 123 (74%) women with a median age of 56 years (range 25‐83), were included in the analyses. Median follow‐up was 15 months (interquartile range 6‐45). Patients and treatment characteristics are presented in Table 1. Indications for the psoas hitch procedure were mostly due to complications related to gynecological surgery or general surgery. Another common indication for the psoas hitch procedure was fibrosis, due to radiation therapy, surgery or endometriosis. Presenting symptoms of patients who underwent an elective psoas hitch procedure were urinary incontinence, (recurrent) urinary tract infection, urosepsis, pyelonephritis, abdominal pain, hydronephrosis, and flank pain.

**TABLE 1:** Patient and treatment characteristics

Demographic data	
Age median (range in years)	56 (25‐83)
Gender *N* (%)	
Male	43 (26%)
Female	123 (74%)
Etiology *N* (%)	
Complications of gynacological surgery	51 (31%)
Complications of general surgery	55 (33%)
Complications of urology	9 (5%)
Carcinoma	7 (4%)
Fistulae	8 (5%)
Radiation fibrosis	14 (8%)
Fibrosis (no radiotherapy)	15 (9%)
Trauma	1 (1%)
Other	6 (4%)
Laterality N (%)	
Left	85 (51%)
Right	77 (47%)
Bilateral	4 (2%)
Pre‐operative serum creatinine µmol/L median (interquartile range)	75 (27)
Pre‐operative creatinine clearance mL/min median (interquartile range)	86 (42)
Previous procedures N (%)	
No previous procedure	91 (55%)
Conservative treatment	59 (36%)
Endoscopic treatment	10 (7%)
Complex surgical procedure	3 (2%)

### 3.2 Post‐operative results and complications

Mean post‐operative renal function did not significantly differ from pre‐operative renal function. The mean difference in serum creatinine pre‐ and post‐operative was −9 µmol/L(SD 87, *P* = .3). Estimated mean renal function pre‐ and post‐operative differed by 0.3 µmol/L (SD 23, *P* = .9). Peri‐operative complications were seen in 17 (10%) patients and consisted of damage to the serosa of the small intestine and damaged blood vessels: a small defect of the vena cava, arterial bleeding and bleeding of the epigastric vessels; these were all intraoperatively and uneventfully repaired. One patient suffered from anaphylactic shock following prophylactic antibiotics. Acute complications (<30 days) were found in 72 (43%) patients. Urinary leakage at the site of the anastomosis was seen on imaging in 9 (5%) patients. Table 2 presents the acute complications according to the Clavien‐Dindo classification. In one patient, a grade IV complication occurred and another patient died five days post‐operative due to aspiration pneumonia following gastric retention. Long‐term post‐operative complications (>30 days) are presented in Table 2. Of the 166 patients, 46 (28%) patients died of reasons not related to the psoas hitch procedure, but of progression of their malignancy.

**TABLE 2:** Acute and post‐operative complications

Acute complications (Clavien – Dindo, <30 days) *N (%)*	
Total	166
0	95 (57)
I	43 (26)
II	4 (2)
IIIa	13 (8)
IIIb	9 (5)
IV	1 (1)
V	1 (1)

Long‐term complications (>30 days) *N (%)*	
Recurrent urinary tract infections	15 (9)
LUTS	23 (14)
Kidney failure	2 (1)
Recurrent tumour (urological, gynaecological, intestinal)	6 (4)

**FIGURE 3:**
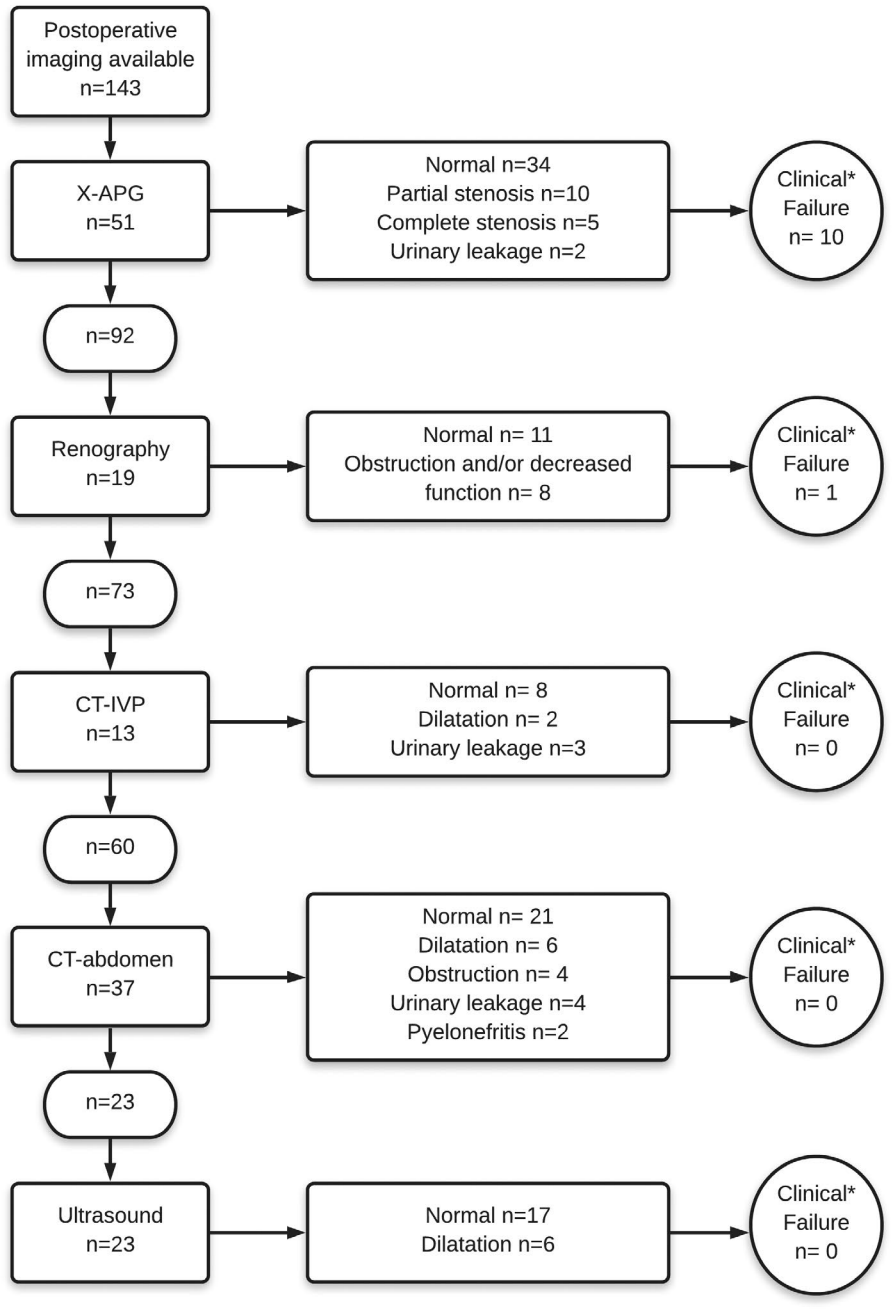
Descriptive of failures based on hierarchal imaging data and clinical information. *Clinical failure was defined as post‐operative obstructive complaints requiring additional treatment

Post‐operative anatomical imaging and/or functional imaging was available in 143 (96%) patients. In 70 (42%) patients, X‐APG and/or renography were performed to assess the passage of the ureteroneocystostomy. Other imaging types were renography, CT‐IVP, CT‐abdomen, and ultrasound. Figure 3 shows the clinical failures, based on available post‐operative imaging and clinical information. In at least 11 of the 143 patients (8%), the psoas hitch was considered a failure. Indications for their psoas hitch procedures were: radiation fibrosis (n = 6), fibrosis due to previous surgery (n = 1), iatrogenic damage during urologic surgery (n = 2), and iatrogenic damage during general surgery (n = 2), see Table 3. Seven of these patients were treated with a double‐J catheter, PCN, or balloon dilatation to improve the passage of the reimplanted ureter. One of these patients showed no improvement in renal function after surgery and his PCN was never removed; this patient died shortly after surgery due to a malignant underlying disease. Three patients were treated with secondary surgery; one patient underwent a Boari‐flap procedure to restore continuity of the ureterovesical junction and in the other two patients an ileal conduit was created. A very small group of nine patients performed a micturition diary for 3 days. The mean functional capacity was 471 mL (250‐810 mL).

**TABLE 3:** Indications for psoas hitch procedure in failures

Indication for psoas hitch procedure	Number of psoas hitch failures Total = 11 (100%)
Radiation fibrosis	6 (55%)
Fibrosis due to previous abdominal surgery	1 (9%)
Iatrogenic damage during urologic surgery	2 (18%)
Iatrogenic damage during general surgery	2 (18%)

## 4 DISCUSSION

Worldwide laparoscopy is overtaking open surgery in many procedures. The present study analyzed a large multicenter cohort of open psoas hitch procedures to gain greater insight into the long‐term complications. In the literature, several small case series on the surgical success of the psoas hitch procedure already exist.^5^, ^7^, ^8^, ^9^, ^10^, ^11^, ^12^, ^13^, ^16^ Furthermore, two large retrospective cohort studies are available. In 1984, Riedmiller et al. and Staehler et al. presented their results on 181 and 110 patients, with a mean follow‐up of 4.5 years and a median follow‐up of 17 months, respectively.^14^, ^15^ However, both studies included a large number of children in their cohorts. Post‐operative results in children and adults are expected to be different, taking into consideration underlying diseases requiring previous treatments with associated complications (i.e., radiotherapy in cancer patients or endometriosis). Riedmiller et al. reported a surgical success rate of 96.7%, for a group consisting of both adults and children. In their study, the psoas hitch procedure was performed in 79 adult patients. They reported six failures of the psoas hitch procedure. Unfortunately, it is not possible to determine whether these failures occurred in children or adults. In addition, Riedmiller et al. did not give details about the post‐operative follow‐up.^14^ In the study by Staehler et al., it was possible to identify the number of psoas hitch procedures performed in adult patients (n = 76), resulting in a surgical success (according to their criteria) of 67%.^15^ Considering this available literature, the present study is the largest retrospective cohort study addressing the long‐term results of the open psoas hitch procedure in adult patients.

While being the largest study so far, our study also has limitations. After finalizing the data collection, it became clear that due to incomplete follow‐up there was insufficient data to define the clinical failure of the psoas hitch procedure in some patients. Since both participating centers are academic hospitals, it is likely that after complex surgical procedures, patients were referred back to their peripheral hospitals for follow‐up, resulting in incomplete data in the electronic patients’ files of the academic hospitals. Therefore, the failure of the psoas hitch procedure of 8% (11/143) should be interpreted with caution as this might be higher. Despite the unsatisfactory data, the precautious assumption can be made that as patients have not returned to the academic hospital with post‐operative urological complaints, the procedure appears to have been effective, with few severe complications and with only a failure rate of (at least) 8%. Notably, we believe that the postoperative complications are predominantly caused by the underlying morbidities and primary surgical interventions and not by the psoas hitch procedure itself.

Due to the retrospective nature of this study, information is missing on the size of the bridged ureteral defects and the characteristics of the stenosis (partial, complete). Previous radiotherapy generally results in impaired wound healing,^21^ this could explain why 6 of the 11 failures were patients with radiation fibrosis. Therefore, caution should be taken when patients previously treated with radiotherapy in the abdominal region undergo a psoas hitch procedure to restore continuity of the ureter. The decision to perform a psoas hitch procedure or a (conservative) alternative procedure in patients with a history of pelvic irradiation should be made at the discretion of the urologist.

The psoas hitch procedure has so far been performed predominantly with an open approach. However, case series ranging from 9 to 18 patients,^22^, ^23^, ^24^, ^25^, ^26^, ^27^, ^28^, ^29^ and one prospective cohort study of endometriosis patients (psoas hitch n = 94),^30^ reported on the feasibility of laparoscopic and robot‐assisted psoas hitch procedures with outcomes comparable to open surgery. For future research on psoas hitch procedures performed with a laparoscopic or robotic approach, our advice is to carry out effective follow‐up and documentation of patients in whom a psoas hitch is performed, in order to be better able to assess the long‐term results of the psoas hitch procedure.

To the best of our knowledge, this study describes the largest retrospective cohort of adult patients in the pre‐laparoscopy era and provides greater insight into the long‐term complications of the open psoas hitch procedure. In conclusion, the psoas hitch procedure is a relatively safe and effective procedure to restore the continuity of the ureter with preservation of kidney function in case of different types of ureteral pathologies. Since radiation fibrosis is the most common cause of clinical failure of the psoas hitch procedure, patients suffering from this etiology should be well counseled before the procedure. Conservative options, such as a permanent double‐J catheter or PCN, might be appropriate alternatives.

## CONFLICT OF INTEREST

We declare no competing interests.
